# CORONIS - International study of caesarean section surgical techniques: the follow-up study

**DOI:** 10.1186/1471-2393-13-215

**Published:** 2013-11-21

**Authors:** The CORONIS Collaborative Group

**Affiliations:** 1 The Institute for Women’s Health, London and National Perinatal Epidemiology Unit CTU, Oxford, UK

## Abstract

**Background:**

The CORONIS Trial was a 2×2×2×2×2 non-regular, fractional, factorial trial of five pairs of alternative caesarean section surgical techniques on a range of short-term outcomes, the primary outcome being a composite of maternal death or infectious morbidity. The consequences of different surgical techniques on longer term outcomes have not been well assessed in previous studies. Such outcomes include those related to subsequent pregnancy: mode of delivery; abnormal placentation (e.g. accreta); postpartum hysterectomy, as well as longer term pelvic problems: pain, urinary problems, infertility. The Coronis Follow-up Study aims to measure and compare the incidence of these outcomes between the randomised groups at around three years after women participated in the CORONIS Trial.

**Methods/Design:**

This study will assess the following null hypotheses: In women who underwent delivery by caesarean section, no differences will be detected with respect to a range of long-term outcomes when comparing the following five pairs of alternative surgical techniques evaluated in the CORONIS Trial:

1. Blunt versus sharp abdominal entry

2. Exteriorisation of the uterus for repair versus intra-abdominal repair

3. Single versus double layer closure of the uterus

4. Closure versus non-closure of the peritoneum (pelvic and parietal)

5. Chromic catgut versus Polyglactin-910 for uterine repair

The outcomes will include (1) women’s health: pelvic pain; dysmenorrhoea; deep dyspareunia; urinary symptoms; laparoscopy; hysterectomy; tubal/ovarian surgery; abdominal hernias; bowel obstruction; infertility; death. (2) Outcomes of subsequent pregnancies: inter-pregnancy interval; pregnancy outcome; gestation at delivery; mode of delivery; pregnancy complications; surgery during or following delivery.

**Discussion:**

The results of this follow-up study will have importance for all pregnant women and for health professionals who provide care for pregnant women. Although the results will have been collected in seven countries with limited health care resources (Argentina, Chile, Ghana, India, Kenya, Pakistan, Sudan) any differences in outcomes associated with different surgical techniques are likely to be generalisable throughout the world.

**Trial registration:**

ISRCTN31089967

## Background

Caesarean section is one of the most commonly performed operations worldwide and accounts for up to 60% of deliveries in some countries, with a rate of approximately 24.6% in the UK in 2008/2009 [1]. The operation is not performed in a standardised way, and there are variations in the surgical techniques used [2]. Caesarean section carries a risk of short-term post-operative morbidity, for example, fever, pain and postpartum haemorrhage. Also of major importance are the long-term clinical and obstetric problems such as chronic pain, infertility, bowel obstruction, abnormal placentation and its consequences, and uterine rupture. The effects of caesarean techniques on these long-term outcomes have not been well assessed in randomised controlled trials to date [3].

The MRC funded CORONIS Trial is a 2×2×2×2×2 fractional, factorial trial of different surgical techniques at caesarean section. The trial is comparing (1) blunt versus sharp abdominal entry; (2) exteriorisation of the uterus for repair versus intra-abdominal repair; (3) single versus double layer closure of the uterus; (4) closure versus non-closure of the peritoneum (pelvic and parietal); (5) chromic catgut versus Polyglactin-910 for uterine repair, on a range of short-term outcomes, the primary outcome being a composite of maternal death or maternal infectious morbidity.

### Summary of existing evidence

Existing systematic reviews of randomised controlled trials have been unable to draw clear conclusions about whether any of the techniques being compared in the CORONIS Trial are to be preferred [4-9]. Optimisation of surgical techniques may be able to reduce both the short-term and the long-term morbidity associated with caesarean section and although differences may be relatively modest, the commonness of the operation means that even small improvements in outcome may result in substantial improvements in health for many thousands of women and considerable cost savings for health services.

Short-term outcomes are the primary outcomes evaluated in most of the trials assessing alternative techniques for caesarean section. These are often surrogate outcomes and are usually only measured up to 6 weeks after the caesarean section. The most common outcomes assessed are:

• operative time and length of postoperative stay

• use of extra haemostatic sutures

• defects in the uterine scar or thickness of the uterine scar

• infection or complications of the wound

• postpartum endometritis

• decrease in postoperative haematocrit

• use of blood transfusion

• fever or postoperative febrile morbidity

• pain

Other more robust neonatal outcomes are rarely collected such as:

• neonatal death

• birth trauma

• admission to neonatal unit

It is clear that the most important differences in terms of maternal outcomes between different surgical techniques may be apparent in the long-term, including the functional integrity of the uterine and abdominal scar during subsequent pregnancies and other long-term post-operative effects such as chronic pelvic pain, infertility, peritoneal and bowel adhesions and obstruction [10]. Even in the absence of short-term effects, there may well be important long-term differences between the randomised groups (a situation not dissimilar to that seen in the MRC funded ORACLE Trial where there were no short-term differences seen [11]. However, in the 7-year follow-up of the children born to women presenting in preterm labour with intact membranes, there was an excess of cerebral palsy in the children of mothers treated with antibiotics compared with those who received placebo [12]). In CORONIS, there is a real possibility that long-term effects may go in the opposite direction to the short-term effects. For example, non-closure of the peritoneum is quicker than closure and may therefore result in less infectious morbidity in the short-term. However, it may also be associated with involuntary infertility because of excessive intraperitoneal adhesion formation.

Therefore long-term outcomes, even if uncommon, are more likely to have a higher impact on morbidity and can occasionally result in life-threatening events or even death. The long-term outcomes assessed in the few existing studies are: fertility (infertility) after caesarean section, chronic pain, dyspareunia, constipation, adhesion formation and uterine rupture [10,13-16]. Some of these outcomes can only be assessed by measuring morbidity in subsequent pregnancies or at subsequent episodes of abdominal surgery. Therefore, trials with large sample sizes are needed to ensure adequate power to draw conclusions about long-term outcomes between compared groups.

A systematic review of the literature has shown that to date there have been only three long-term follow-up studies of the existing randomised trials of caesarean section techniques. One investigated the long-term effects of single versus double layer uterine closure and two studies assessed long- term outcomes of trials comparing closure versus non-closure of the peritoneum in a subsequent pregnancy [10,13,14]. The sample sizes of these follow-up studies are small and included only 18% of the women randomised into the original trials for two of them [10,13] and 51% for the third [14]. In the only study that compared single versus double layer uterine closure, no statistically significant difference was found on the following outcomes: inter-pregnancy interval, vaginal delivery, length of hospital stay, preterm delivery, amnionitis, postpartum endometritis, placental abruption, postpartum haemorrhage, blood transfusion or uterine dehiscence. However, this was only based on a sample size of 145 women out of the 906 randomised in the original trial [13]. From the two follow-up studies of randomised trials comparing closure versus non-closure of the peritoneum (209 women in total), no differences were observed in terms of intra-abdominal adhesions (65 women evaluated out of the 360 originally randomised) [10], abdominal pain, dyspareunia, constipation, urinary symptoms and infertility (144 women included out of the 280 originally randomised) [14].

Given the relatively low incidence of uterine rupture (0.4 to 0.6% for women undergoing a trial of labour versus 0.2% for those having elective repeat caesarean) and dehiscence (1.1%) [17], attempts to detect differences in these outcomes between different surgical techniques with adequate power is challenging. However, the one large observational study that has looked at this outcome is weakened by several potentially interrelated factors [15]. For example, the caesarean technique used changed from mainly double layer closure to mainly single layer closure during the period studied, and the selection criteria for trial of labour may have changed in this time too. Thus women who had single layer closure may have been more likely to have a trial of labour. Well conducted follow-up studies of randomised trials remain the best option to evaluate these outcomes reliably.

There are still very important outcomes that have not been assessed by any randomised trial or observational study, which have major implications for the health of women and their babies. These include for example: mode of delivery of subsequent pregnancy, the risk of placenta accreta and other abnormal placentation, and postpartum hysterectomy.

Placenta accreta is strongly linked to placenta praevia, a condition recognised to increase as the number of previous caesarean sections rises [18-20]. The presence of placenta accreta is associated with major pregnancy complications, including life-threatening maternal haemorrhage, uterine rupture, peripartum hysterectomy [21] and maternal death, as well as complications associated with surgical removal including damage to bladder, ureters and other organs [22]. We know of no randomised trials comparing different techniques for caesarean section assessing this important outcome.

There has been one large observational study of peripartum hysterectomy relating this to previous caesarean section. This study [21], carried out in the UK, collected all cases of peripartum hysterectomy during a 13 month period. There were 318 confirmed cases of peripartum hysterectomy in an estimated 779,955 total births, giving an incidence of 4.1 per 10,000 total births (95% CI 3.6 to 4.5). Three hysterectomies were undertaken electively for management of malignancy with the remaining 315 undertaken for management of haemorrhage. The most commonly reported causes of haemorrhage were uterine atony (53%), morbidly adherent placenta (39%), uterine rupture (8%) and extension of uterine incision at delivery (6%). Compared with controls, women who had had a peripartum hysterectomy had over three times the odds (odds ratio 3.52, 95% CI 2.35 to 5.26) of having a previous caesarean section. This odds ratio increased with the number of previous sections such that women with a peripartum hysterectomy had over eighteen times the odds of having two or more previous sections compared to control women. In this study, two women died, a case fatality of 0.6% (95% CI 0 to 1.5%).

This study did not collect data about the surgical techniques used at previous caesarean section but it does illustrate the evidence that is beginning to emerge about the important longer term morbidity associated with caesarean section.

There are no on-going studies of long-term follow-up of randomised controlled trials of caesarean section that any of the investigators are aware of. The UK CAESAR Trial [23], which was a 2×2×2 factorial trial of (i) single versus double layer uterine closure, (ii) closure versus non-closure of the pelvic peritoneum and (iii) liberal versus restricted use of a sub-sheath drain has finished recruiting and long-term follow-up of this cohort is planned. This trial was also conducted by the National Perinatal Epidemiology Unit (NPEU) and follow-up is planned for 8–11 years after trial recruitment because the inter-pregnancy interval is substantially longer in a UK population than in the settings participating in CORONIS. A prospective meta-analysis of the trial follow-up studies (for single versus double layer uterine closure) is planned.

### The CORONIS Trial (https://www.npeu.ox.ac.uk/coronis)

The objectives of the CORONIS Trial are to determine whether there are any differences in short-term maternal morbidity when comparing five pairs of alternative surgical techniques undertaken during the time of caesarean section. Information was collected at trial entry, immediately following the operation, at discharge from hospital and at six weeks after delivery. The CORONIS Trial protocol has been published and can be downloaded free at http://www.biomedcentral.com/1471-2393/7/24.

### Trial eligibility

Women are eligible for trial entry if they are undergoing delivery by lower segment caesarean section through a transverse abdominal incision, irrespective of fever in labour, gestational age or whether they have a multiple pregnancy.

Women are not eligible if: (i) there is a clear indication for a particular surgical technique or material to be used that prevents any of the allocated interventions being used; (ii) they have had more than one previous caesarean section; (iii) they have already been recruited into the trial during a previous pregnancy.

### Trial primary outcome

Death or maternal infectious morbidity (one or more of the following: antibiotic use for maternal febrile morbidity during postnatal hospital stay, antibiotic use for endometritis, wound infection or peritonitis) or further operative procedures; or blood transfusion (>1 unit of whole blood or packed cells).

### Trial interventions

There are five pairs of interventions being tested in the trial; however, each participating hospital only takes part in three of these five possible comparisons. The non-allocated comparisons and all other aspects of the caesarean section are performed at the discretion of the surgeon.

1. Blunt versus sharp abdominal entry

2. Exteriorisation of the uterus for repair versus intra-abdominal repair

3. Single versus double layer closure of the uterus

4. Closure versus non-closure of the peritoneum (pelvic and parietal)

5. Chromic catgut versus Polyglactin-910 for uterine repair

### Factorial trials

Factorial trials maximise the efficiency of a trial by including more than one trial question into a single trial population. Instead of having one trial which compares, for example, single versus double layer closure of the uterine incision and another trial comparing closure versus non-closure of the peritoneum, both comparisons can be combined into one trial using only the number of women necessary to answer one of these questions in isolation. In the CORONIS Trial, five comparisons have been carried out in one trial. Such a design has rarely been used, but is appropriate for the evaluation of several procedures which will be used together in clinical practice. In such a trial of different caesarean section techniques, using five pairs of possible allocated interventions (1 versus “not 1”, 2 versus “not 2”, 3 vs. versus “not 3”, 4 versus “not 4”, 5 versus “not 5”), participants can receive one of 32 possible alternatives.

In the analysis of a factorial trial the same process is used for a 2×2×2×2×2 factorial design as for a 2×2 factorial design. All those allocated 1 are compared with all those allocated “not 1” regardless of what other interventions were allocated, provided that there is no interaction.

### Fractional factorial trials

In a standard 2×2×2×2×2 factorial design, all participants would receive one of 32 possible alternatives, and each of these alternatives would include five interventions. The CORONIS Trial is a fractional factorial design, and, therefore, each participant was allocated only three out of the five possible interventions. Such an incomplete factorial design was considered appropriate because it allows five surgical techniques to be tested in the same trial, but restricts the number of techniques being tested per centre and per surgeon to three. Hence, each centre was required to implement training for three (not five) surgical techniques and each surgeon was required to remember the three (not five) allocated techniques for every randomised woman.

There were two main implications of this incomplete factorial design. First, in the trial design, we ensured that a similar number of women were included in each of the five comparisons. Second, in the analysis, we will not be able to test for interactions between more than three interventions.

CORONIS recruited in excess of 15,000 women in Argentina, Chile, Ghana, India, Kenya, Pakistan and Sudan. This represents a unique cohort of women to follow up to assess and compare their long-term health outcomes between the different randomised components. In order to ensure a high rate of follow-up while maximising the number of women who will have had a subsequent pregnancy, we have chosen to follow up this cohort at least three years after their participation in CORONIS. Any longer and we risk losing women to follow-up, any shorter and we decrease the number of women who will have had a subsequent pregnancy and therefore limit our power to address important potential differences in outcome.

## Methods/Design

### The CORONIS Follow-up study

#### Objective

The objective of the CORONIS Follow-up Study is to measure and compare the incidence of outcomes at around three years between the randomised groups of women who participated in the CORONIS Trial.

#### Hypothesis

This study will assess the following null hypotheses: In women who underwent delivery by caesarean section, no differences will be detected with respect to a range of long-term outcomes when comparing the following five pairs of alternative surgical techniques:

1. Blunt versus sharp abdominal entry

2. Exteriorisation of the uterus for repair versus intra-abdominal repair

3. Single versus double layer closure of the uterus

4. Closure versus non-closure of the peritoneum (pelvic and parietal)

5. Chromic catgut versus Polyglactin-910 for uterine repair

#### Tracing women

All women participating in the CORONIS Trial have been informed that we hope to be able to interview them at least three years after they were included in CORONIS to ask them about their long-term health.

The trial entry information leaflet contains the following paragraph:

“*We are also keen to find out whether the different methods used make any difference to your long*-*term health. We would like to contact you again after the study has finished. We will ask you how you are and whether you have had any more babies*.”

In addition, at the ‘six week’ follow-up appointment, women are provided with the following letter:

“*Thank you for taking part in CORONIS and for letting us know about your health since your caesarean section*.”

*We hope to see all women who take part in the study once more. This will be three years after the caesarean section. At this visit we will also ask about your health since your caesarean section. Because a lot can happen in three years*, *we will keep in touch with you every 6 months or so to make sure we have your correct phone number and address*.

*If you have any more pregnancies or have any surgery it would be very helpful if you*, *or your doctor*, *could write these down on the CORONIS Medical Card. This will make it easier for you to remember everything that has happened since your caesarean section*.

*If you move house or change your phone number*, *please fill in the Change of Address card and send it back to the Study Office*, *or phone us. The phone number and address are*: [*details*]

“*If you do not want to take part in the study any more*, *you can let us know by phoning or writing to the Study Office. Thank you for your help with this study*.”

To facilitate long-term follow-up women have been provided with a medical card and a document bag for storing their medical information. This bag has the CORONIS Trial logo printed on the outside and contains information about letting the local Regional Trial Office know if the woman moves house or changes other contact details.

The process of maintaining contact with women who participated in CORONIS between their six week follow-up and the three year follow-up is coordinated by each Regional Trial Office to reflect differences in the circumstances between the very different settings for this trial.

#### Outcomes

The list below represents the full range of outcomes relevant to the five intervention pairs being evaluated.

#### Women’s health and mortality

1. Following the CORONIS birth and before any subsequent pregnancy, any new onset or worsening of:

i. pelvic pain

ii. dysmenorrhoea

iii. deep dyspareunia

iv. urinary symptoms of poor stream and/or frequency which did not respond to antibiotics

2. Diagnostic laparoscopy or diagnostic laparotomy (not related to pregnancy)

3. Hysterectomy or tubal/ovarian surgery (not related to pregnancy)

4. Bladder or bowel damage in those women who have had surgery, excluding diagnostic laparoscopy and diagnostic laparotomy (not related to pregnancy).

5. Following the CORONIS birth, any new onset of:

i. abdominal hernia

ii. bowel obstruction

6. Woman’s death

#### Reproductive status

7. Number of women with no subsequent pregnancy

i. Voluntary infertility

ii. Involuntary infertility

8. Use of fertility treatments

#### Subsequent pregnancies

9. Number of women having any subsequent pregnancy and for these women, the following outcomes will be measured:

i. Inter-pregnancy interval from the CORONIS birth to the end of the subsequent pregnancy (regardless of loss or birth)

ii. Miscarriage of the pregnancy subsequent to the CORONIS birth

iii. Ectopic pregnancy

For the birth following the CORONIS birth:

iv. Gestation at delivery (by best estimate) of the first viable pregnancy (gestational age > 24 or >28 weeks depending on country specific definition)

v. Stillbirth

vi. Neonatal death

vii. Mode of delivery:

a. Non-instrumental vaginal

b. Instrumental vaginal

c. Pre-labour caesarean section

d. In labour caesarean section

viii. Other pregnancy complications (one or more of the following components: uterine rupture, uterine scar dehiscence, placenta praevia, morbidly adherent placenta, abruption, postpartum haemorrhage requiring transfusion >1 unit of whole blood or packed cells, severe infection within 6 weeks postpartum, hysterectomy up to 6 weeks postpartum, manual removal of placenta)

ix. Bladder or bowel damage at the time of subsequent caesarean section

#### CORONIS children – morbidity and mortality

10. Death or serious morbidity of the child who was born at the time of CORONIS participation.

Parent report of one or more of the following: child's blindness, deafness, inability to speak or walk without assistance, or other major morbidity such as the presence of a stoma. Although no difference in death is expected, there is likely to be a time difference between ‘sharp’ and ‘blunt’ abdominal entry and this may, in theory, lead to more neonatal encephalopathy which may lead to a greater risk of later death.

All outcomes are potentially relevant for each comparison pair; however, some outcomes are more likely to be influenced by some interventions compared with others. Therefore we have categorised comparisons into (i) main comparisons of interest, and (ii) secondary comparisons of interest (Table 1). This approach has been pre-specified to take account of multiple testing and to simplify reporting of the study. Only the main comparisons of interest will be reported in the main paper, although the remaining comparisons will be presented in appendices to ensure transparency and completeness of the analysis.

**Table 1:** **Comparisons of interest** (**M denotes main comparisons of interest**, **s denotes secondary comparisons of interest**)

**Outcome reference number**	**Outcome**	**Bl vs. Sh**	**Ext vs.-A**	**Sl vs. Dble**	**Cl vs. N-Cl**	**Cat vs. P910**
***Women’s health and mortality***						
1 i)	Pelvic pain	s	s	s	**M**	s
ii)	Dysmenorrhoea	s	s	s	s	s
iii)	Deep dyspareunia	s	s	s	**M**	s
iv)	Urinary symptoms	s	s	s	s	s
2	Diagnostic laparoscopy or diagnostic laparotomy - not related to pregnancy	s	s	s	**M**	s
3	Hysterectomy or tubal/ovarian surgery - not related to pregnancy	s	s	s	**M**	s
4	Bladder or bowel damage – not related to pregnancy	s	s	s	**M**	s
5 i)	Abdominal hernias	**M**			s	
ii)	Bowel obstruction	s	s	s	**M**	s
6	Woman’s death		s	**M**	s	**M**
***Reproductive status***						
7 i)	Women with no subsequent pregnancy – voluntary	s			s	
ii)	Women with no subsequent pregnancy – involuntary	s	**M**	s	**M**	s
8	Fertility treatments	s	**M**	s	**M**	s
***Subsequent pregnancies***						
9	Any subsequent pregnancy	s	s	s	s	s
i)	Interpregnancy interval	s	s	s	s	s
ii)	Miscarriage		s	s		s
iii)	Ectopic pregnancy		**M**	s	**M**	s
iv)	Gestation		s	s		s
v)	Stillbirth		s	**M**		**M**
vi)	Neonatal death		s	**M**		**M**
vii)	Mode of delivery		s	**M**		**M**
viii)	Other pregnancy complications (composite of a-i)		s	**M**		**M**
a	Uterine rupture		s	**M**		**M**
b	Uterine scar dehiscence		s	**M**		**M**
c	Placenta praevia		s	**M**		**M**
d	Morbidly adherent placenta		s	**M**		**M**
e	Abruption		s	**M**		**M**
f	Postpartum haemorrhage requiring transfusion of >1 unit of whole blood or packed cells		s	**M**		**M**
g	Severe infection within 6 weeks postpartum		s	**M**		**M**
h	Hysterectomy up to 6 weeks postpartum		s	**M**		**M**
i	Manual removal of placenta		s	**M**		**M**
ix)	Bladder or bowel damage –at the time of subsequent Caesarean section	s	s	s	s	s
***CORONIS children – morbidity and mortality***						
10	Death or serious morbidity of CORONIS child	**M**				

Key: Bl vs. Sh = Blunt versus sharp abdominal entry, Ext vs. I-A = Exteriorisation of the uterus for repair versus intra-abdominal repair, Sl vs. Dble = Single versus double layer closure of the uterus, Cl vs. N-Cl = Closure versus non-closure of the peritoneum (pelvic and parietal), Cat vs. P910 = Chromic catgut versus Polyglactin-910 for uterine repair.

### Data collection

Most of the information requested will be provided by the woman at face-to-face interview. Many of the outcomes being collected are clinical diagnoses which are made every day by clinicians in these settings. Women will be asked whether they have pelvic pain and pain on intercourse. Whether or not they have sought or received medical care for their pain will be used as a marker of severity. For women who are pregnant at the time of the follow-up, data will be collected once the pregnancy is over.

For the relatively small number of women who have one of the outcomes resulting in hospitalisation (including subsequent pregnancy complications, such as uterine rupture or dehiscence, hysterectomy, placenta praevia, or other morbidity including non-pregnancy related hysterectomy and laparoscopy/laparotomy), consent will be requested from the women to access their hospital notes, so that additional information relating to the incident can be collected from the relevant hospital by the assessment doctors. Although medical records in some settings in these countries may be poor, this mechanism is being used to seek confirmation of events. We will not use these data as the primary source of the outcome data – these come from the women. In addition any relevant laboratory reports and histopathology will be used by the clinicians reporting morbidity to support their diagnosis. We will collect these data, but we do not require these for confirmation of the diagnosis. For example a post-partum hysterectomy histopathology report will not be required to support a diagnosis of a morbidly adherent placenta but will be used as supportive evidence, if it is available.

### Analysis

A detailed Statistical Analysis Plan (SAP) will be developed and agreed by the Study Steering Committee (SSC) before the analysis is undertaken.

### Analysis populations

For the primary analyses, all maternal and child outcomes will be analysed in the groups into which they were randomly allocated e.g. comparing the outcome of all women allocated “blunt abdominal entry” with all those allocated “sharp abdominal entry”, regardless of technique received. Maternal and child outcomes will be analysed for all eligible women correctly recruited for whom data are available.

The outcomes of interest for women who have a subsequent viable pregnancy, such as uterine rupture, will be analysed using two different denominators i.e. women who subsequently have at least one pregnancy and all women randomised. This will take account of the potential for differences in the pregnancy rate between the two arms being compared.

### Descriptive analyses

The flow of participants through each stage of the trial will be illustrated in a CONSORT flowchart [24,25]. This will include the numbers (with percentages) of losses to follow-up over the period of the follow-up study (including reasons, where known) by pair of interventions.

Baseline demographic and clinical characteristics will be described separately for the five intervention pairs in the main trial publication, for those women for whom follow-up data are collected. We will compare the characteristics of such women with those for whom no follow-up assessment was possible using tests of statistical significance, overall and by pair of interventions.

### Primary comparative analyses

The primary analyses will be performed on each of the five pairs of surgical techniques. Outcomes will be summarised by intervention group using appropriate summary statistics (counts and percentages, means and standard deviations, or medians and inter-quartile ranges). Comparisons will be made using relative risks (RR) for dichotomous/categorical outcomes, mean differences for normally distributed continuous outcomes, or median differences for skewed continuous variables (unless the data can be transformed to normality). Analysis of time to event outcomes will employ survival analysis techniques [26].

Multiplicity of statistical testing will be addressed in the detailed statistical analysis plan.

With regard to the analysis of child outcomes, multiple births will be treated as independent events in the primary analysis, in order that relative risks may be presented.

Adjusted analyses will be performed on all comparisons to investigate the impact of minimsation factors ‘In-labour or not in-labour caesarean section’ and ‘number of previous caesarean sections (none or one)’. These will be agreed by the investigators and the Study Steering Committee and pre-specified in the SAP.

Subgroup analyses will be similarly agreed and pre-specified in the statistical analysis plan and results will be presented on forest plots, by intervention pair, wherever appropriate.

The robustness of the results will be examined in sensitivity analyses by using multiple imputation techniques to impute missing 3 year follow-up data, and also restricting analyses to a pre-specified follow-up window. Sensitivity of the effect estimates to adjustments for potential clustering of multiple births will also be examined.

Interactions will be explored using a structured approach. Plausible interactions are difficult to identify and not all can be assessed due to the nature of the trial design. A strategy for the analysis of interactions for the main trial has been agreed by the Trial Steering Committee to prevent the investigators being misled by spurious interactions, given the multiplicity of possible explorations. This strategy will be employed for the primary analysis of the follow-up study i.e. analyses of interactions will only be performed on the main comparisons; and three-way interactions will not be investigated unless there is strong evidence of a two-way interaction in the presence of main effects. The results of the exploration of interactions will be interpreted cautiously together with other evidence such as biological plausibility and consistency (e.g. across countries). Plausible interactions for each outcome will be agreed by the investigators and the Study Steering Committee and pre-specified in the SAP.

### Sample size and power

The original sample size for the main trial was calculated assuming a 15% primary outcome event rate in the control group (80% power, 5% two-sided significance level, ability to detect a relative risk of 0.85, 15% loss to follow-up) which resulted in a total sample size required of ~ 15,000 women (~ 9,000 in each intervention pair). The actual overall event rate observed during the conduct of the trial was found to be around 9%. The sample size was increased to 15,492 (9,296 per intervention pair) to account for the reduction in the event rate and agreed by the TSC.

Assuming an overall response rate of at least 80% for the follow-up [27,28], this will provide us with information from ~12,400 women (or ~7,400 for each intervention pair). If we assume approximately 80% of these women will have had a subsequent pregnancy this will result in ~5,900 women in each intervention pair. This number will be sufficient to detect modest but clinically important differences between any principal comparison for this population.

The statistical power available, based on a fixed sample size, for a range of event rates on a selection of outcomes is high (Table 2). These incidence rate estimates are based on two follow-up studies of randomised trials, both of which were from developed countries where the event rates are likely to be lower than those observed in the countries participating in CORONIS. These studies, therefore, are likely to represent a conservative estimate of the anticipated event rates. One study was following a trial of closure versus non-closure of the peritoneum [12] and the other following single versus double layer closure of the uterine incision^13^. Both studies were small (n = 145 and 144 women) and the estimates of some of the outcomes are therefore imprecise. For example, the estimate of uterine dehiscence is based on 1 event out of 145 women, giving an incidence of 0.69% with a 95% confidence interval of 0.12% to 3.8%. The estimate of dyspareunia is based on 27 events out of 144 women giving an incidence of 19% with a 95% confidence interval of 13% to 26%.

**Table 2:** power to detect a specified difference for a fixed sample size

**Outcome examples**	**Event rate in 1st group e.g. single layer closure**	**Event rate in 2nd group e.g. double layer closure**	**Absolute risk difference**	**Power available to detect this difference**
**For all women (number estimated to be ~7,400)**				
Involuntary infertility	3%	4.5%	1.5%	92%
Subsequent pregnancy	48%	44%	4%	93%
Dyspareunia	19%	16%	3%	92%
**For women who have a subsequent pregnancy (number estimated to be ~5,900)**				
Uterine rupture or dehiscence	4%	5.9%	1.9%	92%
Preterm birth	10%	7.5%	2.5%	93%

The power calculations in Table 2 assume equal numbers in each comparison and a 2-5% level of statistical significance.

## Discussion

The results of the CORONIS Follow-up Study will have importance for all pregnant women and for health professionals who provide care for pregnant women. Although the results will have been collected in seven countries with limited health care resources (Argentina, Chile, Ghana, India, Kenya, Pakistan, Sudan) any differences in outcomes associated with different surgical techniques are likely to be generalisable throughout the world. For example, if non-closure of the peritoneum results in a greater risk of involuntary infertility this finding would be relevant to any country setting.

### Publication and dissemination of results

It is important that the findings of this research are widely disseminated. The dissemination strategy will include publication in high impact peer-reviewed journals and presentations at research conferences around the world. In addition, direct communication with a number of policy making organisations will be undertaken. This will include the UK Departments of Health, the Royal College of Obstetricians and Gynaecologists (who have an increasing presence and influence in producing clinical guidelines for developing countries), the World Health Organisation and the Ministries of Health in each of the participating countries. Consumer organisations with an interest in improving maternity care will also be informed of the study results. Encouraging the rapid incorporation of the results into relevant Cochrane reviews, including the Reproductive Health Library will also assist in dissemination. The Chief Investigator will co-ordinate dissemination of data from this study. To safeguard the scientific integrity of the study, all proposals for subsidiary studies linked to the Follow-up Study should be presented to the Project Management Group for approval. All publications from subsidiary studies using data from the original analyses must be submitted to the Study Steering Committee for review before publication. Data from the study will not be presented in public before the main results are published without the prior consent of the SSC.

The Chief Investigator will co-ordinate dissemination of data from this study. To safeguard the scientific integrity of the study, all proposals for subsidiary studies linked to the Follow-up Study should be presented to the Project Management Group for approval. All publications from subsidiary studies using data from the original analyses must be submitted to the Study Steering Committee for review before publication. Data from the study will not be presented in public before the main results are published without the prior consent of the SSC.

The success of the study depends on a large number of clinicians and participants. For this reason chief credit for the results will not be given to the committees or central organisers but to all who have collaborated and participated in the study.

Authorship at the head of the primary follow-up results paper will take the form ‘The CORONIS Collaborative Group’. This avoids giving undue prominence to any individual. All contributors to the study will be listed at the end of the report, with their contribution to the study identified. This will include all members of the Study Steering Committee, the Project Management Group, the International Co-ordinating Centre, the Regional Trial Offices and local investigators at all participating sites.

Those responsible for other publications reporting specific aspects of the study may wish to utilise a different authorship model, such as “[name], [name] and [name] on behalf of The CORONIS Collaborative Group”.

Decisions about authorship of additional papers will be discussed and agreed by the investigators. Authorship of these papers should follow standard academic rules. The criteria for authorship are:

• Substantial contributions to conception and design, or acquisition of data, or analysis and interpretation of data.

• Drafting the article or revising it critically for important intellectual content

• Final approval of the version to be published.

All authors should fulfill all of the criteria for authorship.

Acquisition of funding, collection of data or general supervision alone does not of itself justify authorship.

Presentations given about CORONIS should include an acknowledgement of the contribution of all the investigators and their organisations and of other collaborators and participants.

### Ethics and research governance

The CORONIS Trial was approved by OXTREC (Oxford Tropical Research Ethics Committee (013–06a), and by the relevant research ethics committees in each of the participating countries and centres. Approval for the follow-up study will require similar approvals. The trial is being conducted according to the MRC Guidelines for Good Clinical Practice. The follow-up study will have similar governance structures.

The CORONIS Trial Steering Committee have agreed to continue as the CORONIS Follow-up Study Steering Committee (SSC). This includes the following independent members:

Professor Jim Neilson (Obstetrician)

Miss Felicity Ashworth (Obstetrician)

Professor Simon Cousens (Statistician)

Dr Debbie Chippington Derrick (Consumer representative)

Professor Manorama Purwar (Obstetrician)

Dr Catriona Waddington (Consumer representative)

The CORONIS Follow-up Study starts after recruitment to the trial finishes. Emerging results from the follow-up study cannot therefore influence recruitment to the trial, however, it is possible that early results from the follow-up study demonstrate clear evidence of benefit or harm for one or more interventions or for particular groups of women within the trial, which would be expected to influence clinical practice and therefore justify early publication of interim findings. Therefore, during the follow-up period, unblinded interim analyses will be supplied, in strict confidence, to the SSC at their annual meeting, together with any other analyses the SSC may request. Prior to their first meeting, the SSC will define criteria for premature release of data similar, in principle, to the stopping guidelines used by Data Monitoring Committees.

Collaborators and all others associated with the study may write through the International Co-ordinating Centre (ICC) to the SSC to draw attention to any concern they may have arising from follow-up interviews with women, or about any other matters that may be relevant.

### International Co-ordinating Centre

The NPEU Clinical Trials Unit at the University of Oxford will be the ICC and will be responsible for the day-to-day running of the on-going trial and the follow-up study.

### Regional Trial Offices and participating centres

Each country has a trial office with responsibility to the Regional Co-ordinator. There are two trial offices in India, one in Delhi and one in Vellore. The trial office is responsible for one or more participating hospitals in their country or region.

Argentina

Hospital Nacional Profesor Alejandro Posadas

Haedo

Buenos Aires

Argentina

Hospital Interzonal General de Agudoa Dr. José Penna

Bahia Blanca

Argentina

Hospital Dr. José María Cullen

Sante Fe

Argentina

Hospital J. B. Iturraspe

Sante Fe

Argentina

Hospital Regional Dr. Ramón Carrillo

Santiago del Estero

Argentina

Chile

Hospital Pontifica Universidad Católica de Chile

Santiago

Chile

Sótero Del Río Hospital

Santiago

Chile

Ghana

Komfo Anokye Teaching Hospital

Kumasi

Ghana

India - (north) Delhi

All India Institute of Medical Sciences (AIIMS)

Ansari Nagar

New Delhi

India

Maulana Azad Medical College & Lok Nayak Hospital

New Delhi

India

Lady Hardinge Medical College & Sucheta Kriplani Hospital

New Delhi

India

University College of Medical Sciences & GTB Hospital

Shahdara

New Delhi

India

India - (south) Vellore

Christian Medical College Hospital

Vellore

India

Kenya

University of Nairobi

Kenyatta National Hospital

Nairobi

Kenya

Pakistan

Fatima Bai Hospital

Business Recorder Road

Karachi

Pakistan

Liaquat National Hospital

Stadium Road

Karachi

Pakistan

Countess of Dufferin Hospital

Hyderabad

Pakistan

Sudan

Soba University Hospital

Alamarat

Khartoum

Sudan

Omdurman Maternity Hospital

Omdurman

Sudan

## Abbreviations

ICC: International Co-ordinating Centre; NPEU: National Perinatal Epidemiology Unit; CTU: Clinical Trials Unit; RTO: Regional Trial Office; SAP: Statistical Analysis Plan; SSC: Study Steering Committee; TSC: Trial Steering Committee.

## Competing interests

The CORONIS Trial Collaborative Group declare that they have no competing interests.

## Authors’ contributions

Members of The CORONIS Collaborative Group were involved in the conception and design of the study and are the Regional Co-ordinators overseeing participating sites in their country. PB, The Institute for Women’s Health, London and the NPEU Clinical Trials Unit, Oxford, drafted the manuscript. All members of The CORONIS Collaborative Group edited the manuscript and read and approved the final manuscript.

## Pre-publication history

The pre-publication history for this paper can be accessed here:

http://www.biomedcentral.com/1471-2393/13/215/prepub
